# Make It SMART: Eliciting and Aligning Goals in Home-Based Primary Care

**DOI:** 10.1093/geroni/igaf122.055

**Published:** 2025-12-31

**Authors:** Pietra Bruni, Tara McBride Afonso, Brian Green, Todd Paul, Cynthia Knight, Cherrylyn Alejo, Laura Moore, Michelle Mlinac

**Affiliations:** VA Boston Health Care System, Boston, Massachusetts, United States; VA Boston Health Care System, Boston, Massachusetts, United States; VA Boston Health Care System, Boston, Massachusetts, United States; Jamaica Plain VA Medical Center, Jamaica Plain, Massachusetts, United States; Jamaica Plain VA Medical Center, Jamaica Plain, Massachusetts, United States; VA Boston Health Care System, Boston, Massachusetts, United States; VA Boston Health Care System, Boston, Massachusetts, United States; VA Boston Healthcare System, Boston, Massachusetts, United States

## Abstract

Home-Based Primary Care teams aim to deliver healthcare services to homebound older adults with complex medical and psychosocial issues. Both patients and providers can become overwhelmed with attempting to manage multiple issues simultaneously. Therefore, identifying outcomes that are meaningful to patients can be facilitated through identification of personalized goals. Utilizing a problem-solving approach was identified as a high competency of the interdisciplinary team. However, problem-solving can contribute to maintenance of the status quo in patient-provider interactions and to loss of enthusiasm for goal setting. As part of VA’s Whole Health model, a multi-stepped quality improvement project was implemented within a home-based primary care team. First, the team implemented team goals for each patient and then once this had become routine process, they elicited patient’s own SMART goals. Achievement of these goals was tracked during care planning meetings, and friendly competition between teamlets to set and meet these goals helped to drive enthusiasm and buy-in. Measurement of meeting both team and patient SMART goals was tracked every 90-days across two years, resulting in the team’s ability to meet their own goals rising from 73% to 88% respectively. Obtaining patient SMART goals was built on training and coaching in motivational interviewing skills, and led to 75% of these patient goals to be met over time. Improvements in SMART goals were identified, with qualitative feedback from the team indicating an appreciation for the visibility of progress, modeling by other team members, and feeling a better connection in clinical work.

